# Surgical Management of Congenital Pseudoarthrosis of the Clavicle: A Review of Current Concepts

**DOI:** 10.7759/cureus.18482

**Published:** 2021-10-04

**Authors:** Abdullah A Alsaeed

**Affiliations:** 1 Department of Surgery, Unaizah College of Medicine and Medical Sciences, Qassim University, Qassim, SAU

**Keywords:** plate, k-wire, bone graft, clavicle, congenital pseudoarthrosis

## Abstract

Nowadays, surgical intervention is an accepted treatment for congenital pseudoarthrosis of the clavicle (CPC). The purpose of this literature review is to evaluate the current body of evidence for methods and outcomes of surgical intervention for CPC. CPC is a rare deformity of the middle third of the clavicle not often identified until three to five years of age, at which time surgery is often recommended. The most common indication for surgery is cosmetic appearance, but other indications include pain, shoulder dysfunction, and prevention of complications later in life. Surgical intervention involves the resection and excision of the pseudoarthrosis, bone grafting (most commonly autologous tissue from the iliac crest), and internal fixation using plates or Kirschner wires (K-wires). Plate fixation tends to have fewer complications and better long-term outcomes. Following surgery, outcomes include satisfaction with cosmetic appearance, decreased pain, and improved shoulder function.

## Introduction and background

Congenital pseudoarthrosis of the clavicle (CPC), which was initially described by Fitzwilliams in 1910 [1] and Owen in 1970 [2], is a rare condition in which there is a failure of fusion between the medial and lateral ossification centers of the clavicle. CPC is present at birth but often not diagnosed until early life (three to five years) as a deformity of the middle third of the clavicle [3]. The prevalence of CPC is rare, with only 200-300 cases reported worldwide [4].

CPC usually occurs in the middle third of the clavicle. Although the exact etiology remains unclear, the theory of how CPC develops is related to clavicle ossification. During gestation, the clavicle is the first bone to ossify [3,5-7]. It begins to appear in the fourth week of gestation and fuses in the seventh week [8]. CPC more often occurs in the right clavicle, with the theory being that pressure from the subclavian artery (oriented more directly underneath the clavicle on the right side) prevents the union of the clavicle [4,5,9-11]. Elevated first and upper ribs may also increase pressure on the subclavian artery. Occurrence on the left side is rare and associated with dextrocardia [11]. Bilateral occurrence is also rare and often associated with bilateral malpositioning of the subclavian artery and upward elevation of the first few ribs [5,9,11,12-14].

CPC is usually discovered early in a child’s life, at around three to five years of age. In a review of studies including 158 patients, the mean age of diagnosis was three years [9]. Cases reported in the literature show female patients to be more often affected [3,4,6].

Careful history taken from the patient and/or parents, clinical examination, and radiological evaluation are essential in making the diagnosis of CPC. The presentation of CPC includes an atraumatic deformity with swelling over the middle third of the clavicle. Shoulder shortening can accompany the deformity, although shoulder function and range of motion are not usually affected. The first rib may also be affected and present with excessive elevation. There have been rare cases of thoracic outlet syndrome and brachial plexus nerve compression associated with CPC [15,16]. The appearance of CPC in radiographs may mimic a traumatic clavicle fracture. In CPC, the borders are bulbous and sclerotic, unlike a clavicle fracture, in which the borders are sharp and non-sclerotic. Moreover, CPC is associated with hypoplastic changes, whereas a clavicle fracture will have no hypoplastic changes with varying degrees of callus formation on follow-up imaging. Other less common differential diagnoses include cleidocranial dysostosis, neurofibromatosis, and short clavicle syndrome [17].

## Review

Surgical indications

Because of the rarity of CPC, comparative studies of surgical versus conservative treatments are few. Conservative treatment is considered when there is no pain or activity restrictions for the child with CPC. Although some patients do well with conservative management [4], others have had worse patient-reported function of the upper extremity [13]. There have also been cases of patients managing CPC conservatively and who later developed thoracic outlet syndrome and vascular complications in adulthood [13,15,16,18-20]. For these reasons, surgical management is a widely accepted treatment of CPC. Patients with CPC often elect to undergo surgery because of pain, the non-aesthetic appearance, and/or the deformity that increases in size as the child ages. Surgery is indicated because the spontaneous union will not occur. Surgery is also indicated to prevent further sequelae from developing in adulthood, as mentioned above.

Timing of surgery

The timing of surgery for patients with CPC is still a debated topic. Many reports point to earlier intervention for more successful outcomes, but parents often elect to delay surgery when their child is very young. Surgery in children at a very young age (<18 months) presents a risk of possible splintering of bone [5], as well as nonunion [13]. On the contrary, nonunion has also been more often reported in patients more than eight years of age [3]. Therefore, surgery should occur in the middle of the childhood years. According to a review of studies up to 2010, operative treatment was most successful in children aged two to four years [9]. More recent reports suggest five to seven years for surgical intervention [21,22], while most of the literature recommends three to five years [9,14,23], indicating that resection of the focus of the pseudoarthrosis occurs at this time [9]. In the largest study to date comparing the timing of surgical intervention by Kim et al., rates of returning to the operating room were greater in patients who underwent surgery at >18 months (median age 6 years), but rates of nonunion were more common in the patients who underwent surgery at <18 months (median age 8 months) [13]. Therefore, the risks should be carefully considered when deciding on the timing of surgical intervention.

It should be noted that surgery can be successfully performed in older patients as well. Adolescent and adult patients do not usually seek surgical intervention for CPC unless it is accompanied by pain, paresthesia, or loss of shoulder function. The oldest patient in the largest study of CPC was 16 years [13]. Two separate case reports on adult patients aged 19 and 21 years have shown relief of symptoms and improved function following surgical intervention [16,24].

Surgical intervention

Surgical intervention involves the resection and excision of pseudoarthrosis, bone grafting, and internal fixation. Bone grafting most often involves the use of autograft tissue from the iliac crest [3,6,12-14,16,21,23,25-28]. Other grafts have included allograft, local corticocancellous graft from debrided pseudarthrosis fragments, and a mix of autograft and allograft [3,13]. Di Gennaro studied patients being treated with bone grafting and found that bone union was achieved in 74% of the cases (n=14/19) [3]. The patients who experienced nonunion were over the age of eight years, and three out of the four patients were treated with fibular allograft. Therefore, the authors did not recommend the use of allograft [3]. While extensive grafting may not be needed for infants or very young children [29], it is certainly recommended in older children (more than eight years of age) and adults undergoing surgery for CPC [16].

The two primary methods of internal fixation involve the use of reconstruction plates [4,12,13,16,21-26,28,30] or K-wires [3,4,6,14,23,27,28]. One alternative method for internal fixation used by Kim et al. for patients younger than 18 months was nonabsorbable suture [13].

Although complications have been reported for the various fixation methods, most studies favor the use of reconstruction plates. Many of the studies using K-wires are older and occurred prior to the routine use of locking plates and anatomic-specific plates found in more recent studies by Kim et al. and Studer et al. [13,22]. On the contrary, Di Gennaro et al. reported preference of K-wire fixation [3]. They abandoned the use of plate fixation after a case of septic nonunion. Chandran et al. compared two stabilization methods and found that patients treated with reconstruction plates experienced faster union rates (100%) and time to union (three months) compared to patients treated with fully threaded pin fixation (nonunion in two patients; time to union six months) [23]. The pin fixation group also experienced greater rates of infection [23]. Infections have also been reported following the use of K-wires [3,14]. Upon removal of K-wire, pin tract infection resolved [14]. In addition to infections, other complications following the use of K-wire include nonunion [3] and re-cutting of K-wire [27]. The study by Persiani et al. did favor K-wire fixation, given lower failure and reoperation rates (only one patient out of eight with K-wire versus four patients out of nine with reconstruction plates experienced surgical failure and required reoperation) [28].

Post-surgical treatment following CPC surgery usually involves immobilization. In very young children, this can involve the use of thoracobrachial cast for 10-12 weeks. In the largest study of CPC, patients were immobilized with a sling and swathe for six weeks [13]. Other studies have reported the use of a sling for four to six weeks postoperatively [25-26]. Overhead and repetitive movements should be delayed for 12 weeks [26].

Outcomes and complications

Bone Union and Success of Surgery

Outcomes following CPC have been good, especially considering the union of the clavicle site is generally slow. Time to union of the pseudoarthrosis ranges from six weeks [14] to three months [23-25] to 8.5 months post-surgery [22]. In one study, the nonunion rate was 19% (5/24 patients), with three patients being in the <18 month category of receiving surgery [13]. Occasionally, surgical revision will be required for cases of nonunion. For patients who presented with nonunion (n=5, 29%), union was ultimately achieved following revision surgery [28]. Haddad et al. used an induced membrane technique in a two-stage procedure to reconstruct the clavicle in three patients (age range seven to nine years) with CPC [27] . Although bone healing and pain-free function were achieved, this technique did result in complications and each patient ultimately underwent three to four total procedures.

Cosmetic Outcomes

Patients have reported satisfaction with the new cosmetic appearance, but it is important to note that the surgical procedure for CPC will result in a scar in place of the previous deformity [14,21,25]. Parents and patients should be made aware of this scar, in addition to the scar that will result from bone grafting.

Pain and Functional Outcomes

In terms of pain and functional outcomes, patients do not often experience long-term issues following surgical intervention [4,13,16,21,22,25,26]. The study by Kim et al. compared surgical versus nonsurgical intervention, and results favored surgery [13]. At the 1.5-year follow-up, the surgical intervention resulted in the resolution of symptoms in all patients who presented with pain and loss of motion prior to surgery [13]. Further, surgical patients reported a perfect score on the Quick Disabilities of the Arm, Shoulder, and Hand (QuickDASH), while nonsurgical patients reported a median score of 11, indicating a higher level of shoulder disability [13]. Although there were no differences in satisfaction of treatment or the Patient-Reported Outcomes Measurement Information System (PROMIS®), the results of this study favor surgical intervention, particularly for younger patients or patients presenting with preoperative pain or loss of function. On the contrary, Persiani et al. reported on two patients who had pain post-surgery when they were previously asymptomatic. Both patients were four years old at the time of initial surgery, treated with plate fixation, experienced complications (one breakage and one mobilization), and required revision surgery with another plate [28].

Most concerning of the pain and functional complications include thoracic outlet syndrome and nerve damage to the brachial plexus [30]. In the case of a patient who had severe thoracic outlet syndrome following conservative management who elected to undergo surgery, there was no pain or functional complications following surgery [16].

Table 1 summarizes the study information of 15 published papers from 1995 to 2021 (each includes at least one surgical case of CPC) that display the CPC characteristics with a clear approach of surgical indications, fixation techniques, and outcomes.

**Table 1:** Literature review and summary CPC = congenital pseudoarthrosis of the clavicle; M = male; F = female; R = right; L = left; B = bilateral; ROM = range of motion; K-wire = Kirschner wire; OR = operation room; QuickDASH = Quick Disabilities of the Arm, Shoulder, and Hand

Author, year	n	Sex	Patient age	Side of CPC	Indication for surgery	Fixation method	Bone graft	Outcomes
Hirata et al., 1995 [21]	1	1 M	5 years	1 R	The patient underwent surgery for cosmetic reasons	Excision of pseudoarthrosis; internal fixation with plate	Autograft from iliac crest	Union achieved at 3 months; plate removed at 6 months
Cadilhac et al., 2000[4]	25	9 M, 16 F	6 years	25 R	17 patients underwent surgery for cosmetic or pain reasons	Excision of pseudoarthrosis; internal fixation with K-wire or plate	Bone graft (unknown type) used in 8 patients	Union achieved: n=14; 2nd surgery: n=3 (no bone grafting); abnormal ROM: n=1; abnormal cosmetic result: n=6
Lorente et al., 2001 [14]	6 (7 clavicles)	6 M	18 months to 4 years	6 R, 1 B	5 patients underwent surgery for cosmetic reasons or avoidance of complications in future	Excision of pseudoarthrosis; internal fixation with K-wire	Autograft from iliac crest	Union achieved in all at 6 weeks; all patients satisfied with cosmesis; superficial pin tract infection: n=3 (resolved once wire removed); no infections at donor site; no pain or abnormal ROM; bone remodeling happened quicker in cases treated earlier
Gomez-Brouchet et al., 2004[6]	5	1 M, 4 F	6.5 years	5 R	All patients underwent surgery for cosmetic or pain reasons	Excision of pseudoarthrosis; internal fixation with K-wire	Autograft from iliac crest	Union achieved: n=4; 1 patient (the older) underwent revision due to recurrence after severe trauma
Ettl et al., 2005[25]	3	1 M, 2 F	6 years	2 R, 1 L	All patients underwent surgery for cosmetic reasons	Excision of pseudoarthrosis; internal fixation with plate	Autograft from iliac crest	Union achieved in all at 3 months; all patients satisfied with cosmesis; no pain or abnormal ROM
Persiani et al., 2008[28]	17	8 M, 9 F	6 years	17 R	All patients underwent surgery for cosmetic reasons, defect of shoulder, or prevent further complications	Excision of pseudoarthrosis; internal fixation with plate, n=9; K-wire, n=8	Autograft from iliac crest, n=12; no autograft, n=5	Union achieved after initial surgery, n=12; after revision surgery, n=5; failure with plate fixation, n=4; with K-wire, n=1; asymmetric shoulder girdle: n=5; previously pain-free patients complained of pain: n=2; decreased muscle strength: n=1; shorter clavicle: n=5; good result: n=11 (4 plate and 7 K-wire); fair result: n=3 (2 plate and 1 K-wire); poor result: n=3 (3 plate)
Chandran et al., 2011 [23]	10	Not stated	5 years	9 R, 1 B	Not stated	Excision of pseudoarthrosis; internal fixation with plate, n=5; K-wire, n=5	Autograft from iliac crest	K-wire fixation: Union achieved at 6 months; infection: n=1; plate fixarion: union achieved at 3 months; prominent metal that had to be removed: n=1; all patients were pain-free and had full ROM
Galanopoulos et al., 2012[26]	1	1 M	9 years	1 R	The patient underwent surgery for cosmetic reasons	Excision of pseudoarthrosis; internal fixation with plate	Autograft from pseudoarthrosis fragments	Union achieved; no pain; normal ROM
Watson et al., 2013 [16]	1	1 F	19 years	1 R	The patient had only swelling with no pain at age of 7 years (conservative); however, the patient presented again at the age of 19 with pain and neurovascular symptoms	Excision of pseudoarthrosis; internal fixation with plate	Autograft from iliac crest	Union achieved; symptoms resolved and functional recovery was achieved
Magu et al., 2014[24]	1	1 F	21 years	1 R	The patient underwent surgery due to pain with overhead activity	Excision of pseudoarthrosis; internal fixation with plate (this was a case of bifurcation of the CPC)	Autograft from iliac crest	Union achieved; symptoms resolved
Di Gennaro et al., 2017 [3]	27	12 M, 15 F	8.1±2.7 years	26 R, 1 L	19 patients underwent surgery for cosmetic reasons	Excision of pseudoarthrosis; internal fixation with K-wire, n=18; plate, n=1	Autograft from iliac crest, n=15; allograft from fibula, n=4	Nonunion: n=5; infection: n=1 (wound debridement and hardware removal); breakage of K-wire: n=1; pin tract infection: n=1; use of allograft was associated with the recurrence of nonunion
Studer et al., 2017[22]	7 (8 clavicles)	3 M, 4 F	7.1 years	6 R, 1 B	All patients underwent surgery for cosmetic or pain reasons	Excision of pseudoarthrosis Internal fixation with plate	Autograft from iliac crest	No wound complications, hardware failures, or late fractures; severe pain at the donor site, but by the 6-week postoperative review, none had donor-site morbidity: n=3; at the 6- and 12-month follow-up, all the children had good clinical function (Constant Score = 94 [88–98 points]); 1-year post-metal removal, satisfactory ROM and functional outcome results
Giwnewer et al., 2018[21]	3	2 M, 1 F	5.7 years	3 R	All patients underwent surgery for cosmetic reasons	Excision of pseudoarthrosis; internal fixation with plate	Autograft from iliac crest	All satisfied and achieved union with no restriction of shoulder ROM; at 1-month follow-up, plate removed due to skin irritation: n=1
Kim et al., 2020 [13]	47	25 M, 22 F	2 years (median)	44 R, 1 L, 2 B	24 patients underwent surgery for cosmetic, pain reasons or limited ROM	Excision of pseudoarthrosis; patients <18 months: n=9, internal fixation with nonabsorbable suture; patients >18 months: n=15, internal fixation with plate	Patients <18 months: local corticocancellous graft from the pseudarthrosis site, n=5; allograft, n=1; patients >18 months: data on bone graft available for 14/15 patients; autologous corticocancellous graft from iliac crest, n=7; local corticocancellous graft from the debrided pseudarthrosis fragments, n=4; combination of local autograft and allograft, n=3	All patients had no pain or ROM restrictions; patients <18 months: nonunion, n=3; no return to OR; patients >18 months: nonunion, n=2; 8 return to OR, hardware removal (n=7) and nonunion (n=1); patients with surgery had better upper extremity function on QuickDASH compared to nonsurgical patients
Martínez-Aznar et al., 2021 [31]	9	5 M, 4 F	4.43 years	9 R	All patients underwent surgery for cosmetic reasons or discomfort	Excision of pseudoarthrosis; internal fixation with K-wire, n=2; plate, n=6		Delayed union, n=1 (plate)

## Conclusions

The most common reasons for surgical intervention are cosmesis followed by pain. Surgical intervention involves pseudoarthrosis resection, bone grafting, and internal fixation. Autografts, commonly from the iliac crest, are most often used due to better union rates and decreased complications; however, allografts and remnants from the pseudoarthrosis can also be used. The current body of literature supports the use of plate over K-wire fixation methods, likely due to limitations of K-wire fixation (nonunion, infection, re-cutting of K-wire). It should be noted that complications can occur with any surgical procedure. Surgical intervention for CPC results in favorable outcomes, including satisfaction with cosmetic appearance, decreased pain, and improved shoulder motion and function.
